# Similia Similibus?

**Published:** 1890-01

**Authors:** 


### IS IT SIMILIA SIMILIBUS?

The following, from the Austin Statesman, is, we believe, the first application of the proposition seriously made some time ago to pit one kind of microbe against another, as a therapeutic measure:

"The editorial staff of the Statesman was suffering all day yesterday, with something that bordered distressingly near the ragged edge of la grippe. The managing editor was a picture of woe, and his cheerfulness was of that character usually found within the precincts of a morgue.

"The grip, or whatever you please to call it, essayed to take in the pious city editor at one fell swoop, and provoked sneezing, prolonged and of all varieties. For a time it was fun, and the Russian visitor appeared to be elated. But his joy was of short duration. After taking possession of the nasal apartments and making himself hilariously comfortable, he started on a voyage of discovery down the bronchial tubes.

"The city editor was 'karm.'

"Lurking in among his bronchial tubes is a very healthy, full-grown and vigorous case of asthma, which has baffled the doctors for years, and caused them to wish themselves dead.

"Just about the time Grip started on his voyage of discovery, the old sinner, Asthma, was preparing for a picnic, and his orchestra, which always accompanies a fashionable and dignified case of asthma, were perching themselves around on the bronchial branches, and tuning their instruments.

At such times he brooks no interference, and grasping Grip by the throat, he calmly wiped up the floor with him, spit in his ear, kicked him in his stomach, and pitched him back into the nasal apartments, where he packed his sneezing apparatus and incontinently made off.

"Russian Grip cannot monkey with American Asthma."

